# A population-based investigation: How to identify high-risk T1-2N0 esophageal cancer patients?

**DOI:** 10.3389/fsurg.2022.1003487

**Published:** 2023-01-17

**Authors:** Peng Luo, Jie Wu, Xiankai Chen, Yafan Yang, Ruixiang Zhang, Xiuzhu Qi, Yin Li

**Affiliations:** ^1^ Department of Thoracic Surgery, National Cancer Center, National Clinical Research Center for Cancer, Cancer Hospital, Chinese Academy of Medical Sciences and Peking Union Medical College, Beijing, China; ^2^ Department of Urology, National Cancer Center, National Clinical Research Center for Cancer, Cancer Hospital, Chinese Academy of Medical Sciences and Peking Union Medical College, Beijing, China; ^3^ Department of Ultrasound, Fudan University Shanghai Cancer Center, Shanghai, China; ^4^ Department of Oncology, Shanghai Medical College, Fudan University, Shanghai, China

**Keywords:** T1-2N0 esophageal cancer, distant metastases, risk factors, nomogram, SEER

## Abstract

**Purpose:**

Newly diagnosed T1-2N0 esophageal cancer (EC) is generally deemed as early local disease, with distant metastases (DM) easily overlooked. This retrospective study aimed to describe the metastatic patterns, identify risk factors and established a risk prediction model for DM in T1-2N0 EC patients.

**Methods:**

A total of 4623 T1-2N0 EC patients were identified in the Surveillance, Epidemiology and End Results (SEER) database from 2004 to 2018. Multivariable logistic regression was used to identify risk factors for DM. A nomogram was developed for presentation of the final model.

**Results:**

Of 4623 T1-2N0 patients, 4062 (87.9%) had M0 disease and 561 (12.1%) had M1 disease. The most common metastatic site was liver (*n* = 156, 47.3%), followed by lung (*n* = 89, 27.0%), bone (*n* = 70, 21.2%) and brain (*n* = 15, 4.5%). Variables independently associated with DM included age at diagnosis, gender, tumor grade, primary site, tumor size and T stage. A nomogram based on the variables had a good predictive accuracy (area under the curve: 0.750). Independent risk factors for bone metastases (BoM), brain metastases (BrM), liver metastases (LiM) and lung metastases (LuM) were identified, respectively.

**Conclusions:**

We identified independent predictive factors for DM, as well as for BoM, BrM, LiM and LuM. Above all, a practical and convenient nomogram with a great accuracy to predict DM probability for T1-2N0 EC patients was established.

## Introduction

With the growth speed greater than that of other dominating epithelial carcinomas, esophageal cancer (EC) ranks as the eighth-most common carcinoma and the sixth-most common cause of death worldwide, indicating that the overall prognosis remains dismal (1–3). Appropriate treatment is critical to patients’ outcome.

For newly diagnosed EC, the choice of initial treatment is largely dependent on clinical stage. According to NCCN guidelines, only for patients with T1b-2N0M0 disease, is esophagectomy chosen as initial treatment (4). T1-2N0 EC is generally deemed as early local disease. Therefore, distant metastases, such as bone and brain metastases, can be easily neglected if doctors relaxed their vigilance. However, DM is not rare among these patients (5, 6). Quint LE et al. have reported a metastatic rate of 18% in newly diagnosed EC using a cohort of 838 from University of Michigan Medical Center (6). Yet it is worth noting that only 75% patients get DM detected before surgery, meaning that up to 25% patients have received futile surgical treatments (6).

Once esophagectomy is carried out on a T1-2N0M1 patient, the timing of palliative management such as chemotherapy will be seriously delayed, severely jeopardizing patients’ outcome and greatly increasing unnecessary treatment expenses since esophagectomy is an extensive and complex procedure with a high incidence of postoperative morbidity and mortality (7).

Positron emission tomography-CT (PET-CT) is deemed as an effective method of identifying patients with occult metastases (8). However, given the relatively high cost, as well as the relatively low incidence of DM in early local EC, in economically underdeveloped countries, PET-CT has not been widely applied to patients of lower socioeconomic status, who are exactly more predisposed to EC (9). For T1-2N0 EC patients, if we can identify those with higher DM probability, the imaging could be carried out more cost-effectively. As a result, predicting factors identification and the prediction model establishment of DM among T1-2N0 EC patient are of great practical meaning, especially to economically underdeveloped countries. However, existing literatures concerning this aspect remain a blank.

In this study, we aimed to describe the metastatic patterns, recognize independent risk factors, develop and validate a nomogram for DM in T1-2N0 EC patients utilizing a cohort from the Surveillance, Epidemiology, and End Results (SEER) public-access database. Besides, independent risk factors for bone metastases (BoM), brain metastases (BrM), liver metastases (LiM) and lung metastases (LuM) among T1-2N0 EC patients were explored, respectively.

## Methods

### Patient selection and data collection

**Figure 1:**
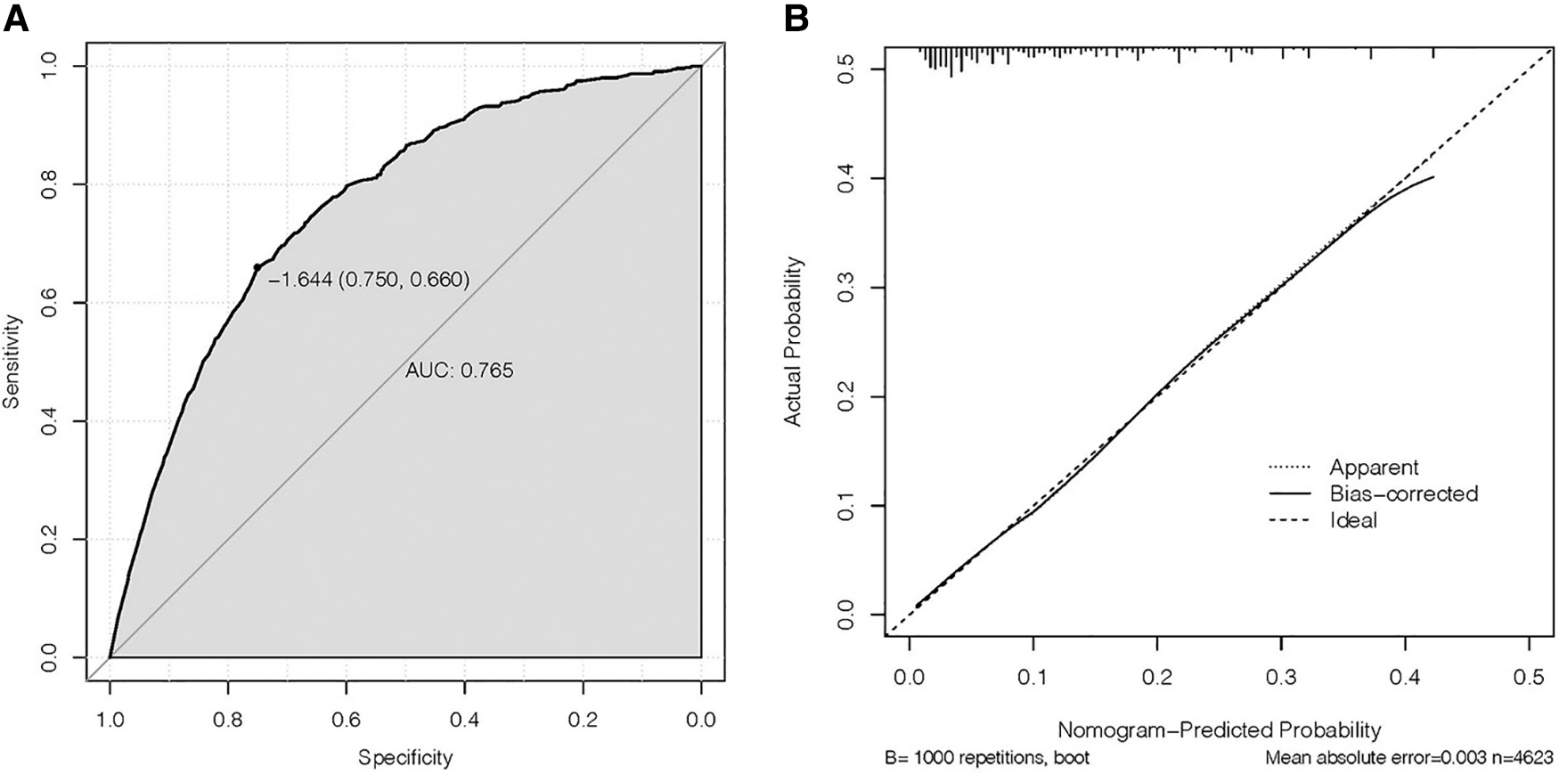
Research flowchart.

This research selected eligible patients from the SEER public-access database for esophageal cancer. Based on the histology code of 8050–8089 (squamous cell neoplasms) and 8140–8389 (adenomas and adenocarcinomas), a total of 48029 cases, diagnosed as esophageal squamous-cell carcinoma (SCC) and esophageal adenocarcinoma (AC) from 2004 to 2018 were obtained from the database. The exclusion criteria were as follows (1): T0, Tis, T3, T4, or regional lymph node metastases (N+) (2), T (primary tumor), N (regional lymph node), or distant metastases of unknown status (3), tumor site, tumor grade or tumor size of unknown status. The flow chart of case selection was presented in Figure 1. Information of interest encompassed gender, age at diagnosis, race, the American Joint Commission on Cancer (AJCC) TNM Staging, year of initial diagnosis, tumor grade, tumor site, tumor size, tumor grade, histology, metastatic organs.

### Statistical analysis

Demographic and clinical characteristics were summarized and presented by count and percentage. Univariable analysis was conducted to identify variables associated with DM. Statistically significant (*P* < 0.05) variables were incorporated into multivariable logistic regression to identify predictors and build the risk model for DM. The information about metastatic status of bone, brain, liver and lung was recorded in SEER database since 2010. Therefore, the metastatic pattern was described based on the data of BoM, BrM, LiM and LuM using the cohort from 2010 to 2018. We further explored the risk factors for BoM, BrM, LiM, and LuM, respectively. All tests were two-sided with a significance level set at *P* < 0.05. Nomograms for risk factors associated DM were established by R software, and the discrimination and accuracy of the model were validated by 2 methods (1): receiver operating characteristic (ROC) curve, and (2) calibration slope for calibration. Bootstrapping using 1000 repetitions was utilized for internal validation of our final nomogram and to calculate the predictive accuracy.

All analysis were carried out by R software 3.5.2 (R Foundation for Statistical Computing, Vienna, Austria). The “survival” package was applied to conduct logistic regression analysis. The “pROC” package was adopted to plot the ROC curve and identify the area under the curve (AUC). The “rms” package was used to build nomogram and calibration curve.

## Results

### Baseline clinical characteristics

After application of selection criteria, a total of 4623 patients were included in this study: 4062 (87.9%) with T1-2N0M0 disease and 561 (12.1%) with T1-2N0M1 disease. The demographic and tumor characteristics were summarized and presented in Table 1. There were 3559 (77.0%) males and 1064 (23.0%) females, with the median age of 68 (range, 22 to 100) years. White race accounted for 86.3% (*n* = 3991). 2763 (59.8%) cases were diagnosed between 2010 and 2018. Lower esophagus (*n* = 3320, 71.8%) was the most common site, followed by middle esophagus (*n* = 930, 20.1%) and upper esophagus (*n* = 373, 8.1%). AC and SCC accounted for 67.7% (*n* = 3128) and 32.3% (*n* = 1495), respectively. Histologically well differentiated, moderately differentiate, and poorly differentiated EC accounted for 11.2% (*n* = 517), 52.3% (*n* = 2417), and 36.5% (*n* = 1689), respectively. 1922 (41.6%) patients had primary tumor >30 mm. As for T stage, T1 and T2 were 3456 (74.8%) and 1167 (25.2%), respectively.

**Table 1:** Clinicopathological variables of patients with T1-2N0 esophageal cancer.

Variables	Total (*N* = 4623)	M0 (*N* = 4062)	M1 (*N* = 561)
Gender			
Male	3559 (77.0%)	3097 (76.2%)	462 (82.4%)
Female	1064 (23.0%)	965 (23.8%)	99 (17.6%)
Age (years)			
≤60	1122 (24.3%)	943 (23.2%)	179 (31.9%)
60–70	1500 (32.4%)	1339 (33.0%)	161 (28.7%)
70–80	1319 (28.5%)	1161 (28.6%)	158 (28.2%)
>80	682 (14.8%)	619 (15.2%)	63 (11.2%)
Race			
White	3991 (86.3%)	3517 (86.6%)	474 (84.5%)
Black	404 (8.7%)	347 (8.5%)	57 (10.2%)
Other	228 (4.9%)	198 (4.9%)	30 (5.3%)
Diagnosis Year			
2004–2009	1860 (40.2%)	1632 (40.2%)	228 (40.6%)
2010–2018	2763 (59.8%)	2430 (59.8%)	333 (59.4%)
Tumor Site			
Upp	373 (8.1%)	338 (8.3%)	35 (6.2%)
Mid	930 (20.1%)	839 (20.7%)	91 (16.2%)
Low	3320 (71.8%)	2885 (71.0%)	435 (77.5%)
Histology			
AC	3128 (67.7%)	2756 (67.8%)	372 (66.3%)
SCC	1495 (32.3%)	1306 (32.2%)	189 (33.7%)
Tumor Grade			
I	517 (11.2%)	500 (12.3%)	17 (3.0%)
II	2417 (52.3%)	2167 (53.3%)	250 (44.6%)
III	1689 (36.5%)	1395 (34.3%)	294 (52.4%)
Tumor Size (mm)			
≤15	1299 (28.1%)	1271 (31.3%)	28 (5.0%)
15–30	1402 (30.3%)	1277 (31.4%)	125 (22.3%)
>30	1922 (41.6%)	1514 (37.3%)	408 (72.7%)
T Stage			
T1	3456 (74.8%)	2996 (73.8%)	460 (82.0%)
T2	1167 (25.2%)	1066 (26.2%)	101 (18.0%)

SCC, squamous-cell carcinoma; AC, adenocarcinoma; Upp, upper esophagus; Mid, middle esophagus; Low, lower esophagus.

### Metastatic patterns

**Figure 2:**
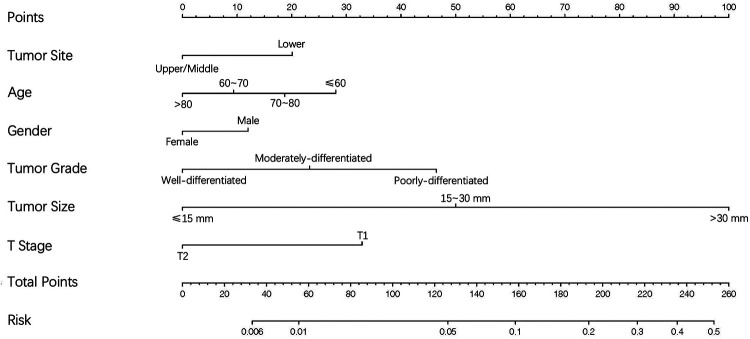
Venn diagram of the distribution of bone metastases, brain metastases, liver metastases and lung metastases from 2010 to 2018.

According to SEER database from 2010 to 2018, 333 patients had initially diagnosed DM. Among these patients, a total of 330 metastases to bone, brain, liver and lung were observed in 248 (74.5%) cases. The distribution of the metastatic site of EC patients were illustrated Figure 2. Within the investigated four metastatic organs, DM was isolated in 181 (73.0%) cases and multiple in 67 (27.0%) cases. The most common metastatic site was liver (*n* = 156, 47.3%), followed by lung (*n* = 89, 27.0%), bone (*n* = 70, 21.2%) and brain (*n* = 15, 4.5%).

### Risk factors identification for dm

Based on univariable and multivariable logistic regression analyses, we finally identified six independent risk factors for DM as follows: age at diagnosis, gender, tumor grade, primary site, tumor size and T stage. The details were provided in Table 2. As for gender, male had a higher risk of DM (OR = 1.33, 95% CI 1.04–1.70, *P* < 0.05). The risk of DM showed a tendency to decrease as age grew, with patients >80 possessing the lowest risks (OR = 0.44, 95%CI 0.32–0.61, *P* < 0.01). Primary site of lower esophagus showed higher risk of DM (OR = 1.61, 95% CI 1.29–2.02, *P* < 0.01) compared to that of upper and middle esophagus. In contrast to well differentiated EC, both moderately differentiated (OR = 2.49, 95% CI 1.49–4.20, *P* < 0.01) and poorly differentiated (OR = 3.90, 95% CI 2.34–6.53, *P* < 0.01) patients had higher risk of DM. In terms of tumor size, an increasing risk of DM was detected as tumor size grew, with size over 30 mm having the highest risk (OR = 12.69, 95% CI 8.53–18.88, *P* < 0.01). As for T stage, T2 showed a lower risk of DM compared to T1 (OR = 0.46, 95% CI 0.36–0.58, *P* < 0.01).

**Table 2:** Univariable and multivariable analyses of variables associated with distant metastases.

Variables	Univariable	*P*	Multivariable	*P*
	OR (95% CI)		OR (95% CI)	
Gender				
Male vs. Female	1.38 (1.19–1.59)	<0.01	1.39 (1.19–1.62)	<0.01
Age (years)				
≤60	(Reference)		(Reference)	
60–70	0.73 (0.63–0.85)	<0.01	0.68 (0.59–0.80)	<0.01
70–80	0.62 (0.54–0.73)	<0.01	0.58 (0.50–0.69)	<0.01
>80	0.47 (0.39–0.57)	<0.01	0.33 (0.27–0.41)	<0.01
Race				
White	(Reference)		–	–
Black	1.19 (1.00–1.42)	0.055	–	–
Other	1.06 (0.81–1.38)	0.685	–	–
Diagnosis Year				
04–09 vs. 10–18	0.97 (0.86–1.08)	0.578	–	–
Tumor Site				
Low vs. Upp/Mid	1.52 (1.31–1.76)	<0.01	1.81 (1.57–2.10)	<0.01
Histology				
AC vs. SCC	1.08 (0.96–1.22)	0.193	–	–
Tumor Grade				
I	(Reference)		(Reference)	
II	2.89 (2.08–4.01)	<0.01	2.10 (1.52–2.92)	<0.01
III	5.34 (3.86–7.39)	<0.01	2.99 (2.15–4.14)	<0.01
Tumor Size (mm)				
≤15	(Reference)		(Reference)	
15–30	4.69 (3.25–6.78)	<0.01	7.63 (5.69–10.22)	<0.01
>30	13.34 (9.46–18.80)	<0.01	22.78 (17.22–30.13)	<0.01
T Stage				
T2 vs. T1	0.66 (0.57–0.77)	<0.01	0.51 (0.43–0.60)	<0.01

OR, odds ratio; 95% CI, 95% confidence intervals; SCC, squamous-cell carcinoma; AC, adenocarcinoma; Upp, upper esophagus; Mid, middle esophagus; Low, lower esophagus.

### Creation and validation of the nomogram

**Figure 3:**
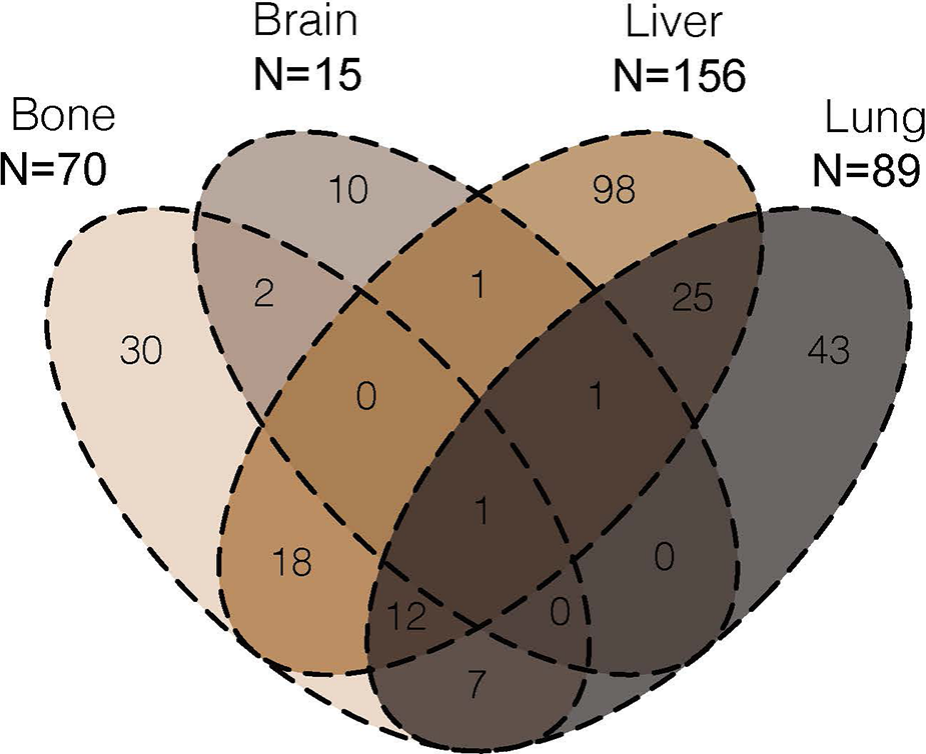
The nomogram for predicting the likelihood of distant metastases in T1-2N0 esophageal cancer.

**Figure 4:**
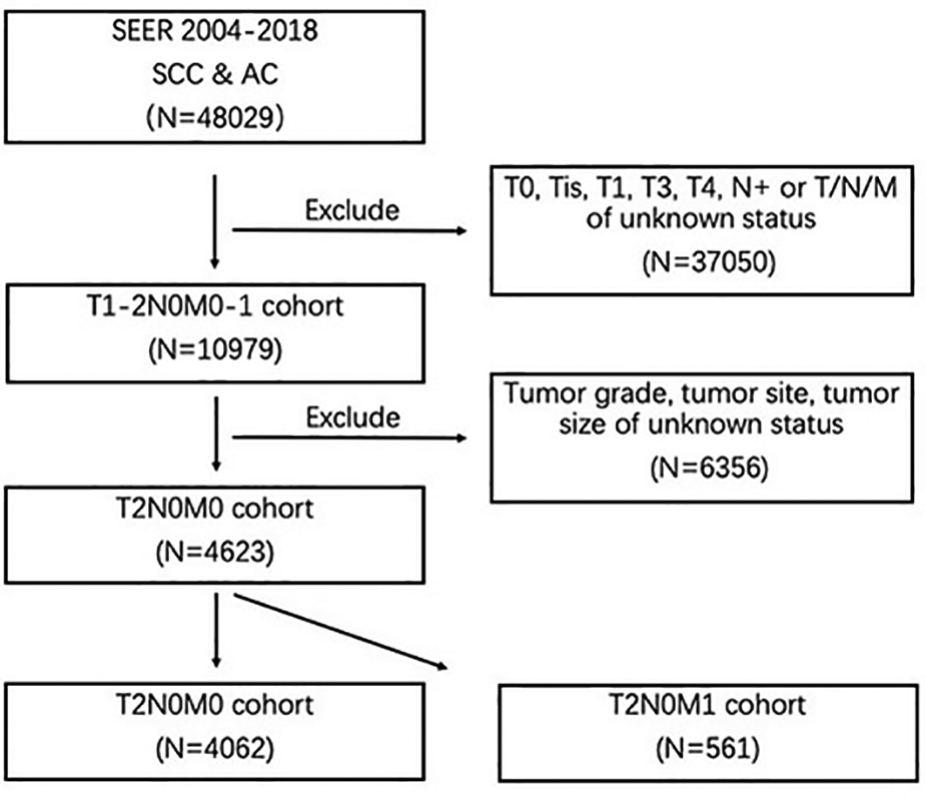
(**A**) receiving operating characteristic (ROC) and (**B**) calibration slope curve of our model.

On the basis of independent risk factors, a nomogram was established to predict DM among T1-2N0 EC patients (Figure 3). Via calculating the score of all the incorporated covariates, the DM probability of the specific patient could be precisely determined. The AUC was 0.750, indicating satisfactory agreement with actual DM probability (Figure 4A). What’s more, the calibration curve for DM suggested perfect consistency between the prediction by our nomogram and the actual DM probability (Figure 4B).

### Risk factors identification for BoM, BrM, LiM and LuM

Using univariable and multivariable logistic analyses, we explored significant risk factors for BoM, BrM, LiM and LuM, respectively. The details of multivariable and univariable logistic analyses were listed in Table 3. Independent risk factors for BoM were younger age, site of lower esophagus, larger tumor size, BrM, LiM, and LuM. Independent risk factors for BrM were BoM, and LuM. As for LiM, independent risk factors included male, younger age, site of lower esophagus, higher tumor grade, larger tumor size, T1 disease, BoM, and LuM. At last, larger tumor size, T1 disease, BoM, and LiM were independently associated with LuM.

**Table 3:** Multivariate analyses of variables associated with bone, brain, liver and lung metastases.

Variables	BoM (*N* = 70)		BrM (*N* = 15)		LiM (*N* = 156)		LuM (*N* = 89)	
	OR (95% CI)	*P*	OR (95% CI)	*P*	OR (95% CI)	*P*	OR (95% CI)	*P*
Gender								
Male vs. Female	2.52 (1.20–5.28)	<0.05	4.49 (0.59–34.19)	0.147	2.72 (1.63–4.54)	<0.01	1.10 (0.67–1.83)	0.703
Age (years)								
≤60	(Reference)		(Reference)		(Reference)		(Reference)	
60–70	0.39 (0.22–0.71)	<0.01	2.28 (0.47–11)	0.306	0.55 (0.37–0.83)	<0.01	0.82 (0.46–1.45)	0.491
70–80	0.42 (0.23–0.78)	<0.01	2.36 (0.47–11.72)	0.295	0.56 (0.37–0.85)	<0.01	0.96 (0.54–1.70)	0.885
>80	0.36 (0.16–0.83)	<0.05	–	0.994	0.38 (0.21–0.69)	<0.01	0.84 (0.41–1.71)	0.623
Race								
White	(Reference)		(Reference)		(Reference)		(Reference)	
Black	0.16 (0.02–1.15)	0.068	–	0.995	0.76 (0.39–1.46)	0.402	2.02 (1.10–3.71)	<0.05
Other	0.87 (0.27–2.82)	0.822	1.38 (0.18–10.59)	0.756	0.82 (0.35–1.89)	0.640	1.37 (0.55–3.47)	0.501
Tumor Site								
Low vs. Upp/Mid	3.28 (1.49–7.19)	<0.01	2.33 (0.52–10.33)	0.267	2.86 (1.74–4.71)	<0.01	0.73 (0.46–1.15)	0.171
Histology								
AC vs. SCC	1.72 (0.95–3.11)	0.071	6.00 (0.79–45.67)	0.084	1.63 (1.10–2.41)	<0.05	0.65 (0.42–1.00)	<0.05
Tumor Grade								
I	(Reference)		(Reference)		(Reference)		(Reference)	
II	2.17 (0.66–7.17)	0.205	–	0.994	2.98 (1.19–7.46)	<0.05	3.52 (1.09–11.4)	<0.05
III	4.85 (1.49–15.79)	<0.01	–	0.994	6.90 (2.78–17.13)	<0.01	5.11 (1.57–16.6)	<0.01
Tumor Size (mm)								
≤15	(Reference)		(Reference)		(Reference)		(Reference)	
15–30	12.36 (1.59–95.96)	<0.05	3.34 (0.35–32.15)	0.297	4.19 (2.14–8.23)	<0.01	11.94 (2.79–51.07)	<0.01
>30	51.06 (7.06–369.37)	<0.01	9.25 (1.19–71.81)	<0.05	8.71 (4.65–16.33)	<0.01	29.25 (7.14–119.76)	<0.01
T Stage								
T2 vs. T1	0.50 (0.26–0.95)	<0.05	0.19 (0.03–1.47)	0.112	0.38 (0.24–0.62)	<0.01	0.46 (0.25–0.82)	<0.01
Metastatic Organ								
Bone	–	–	10.00 (2.76–36.28)	<0.01	16.33 (9.86–27.05)	<0.01	15.21 (8.59–26.93)	<0.01
Brain	10.00 (2.76–36.28)	<0.01	–	–	4.24 (1.18–15.18)	<0.05	4.71 (1.05–21.17)	<0.05
Liver	16.33 (9.86–27.05)	<0.01	4.24 (1.18–15.18)	<0.05	–	–	17.05 (10.78–26.95)	<0.01
Lung	15.21 (8.59–26.93)	<0.01	4.71 (1.05–21.17)	<0.05	17.05 (10.78–26.95)	<0.01	–	–

OR, odds ratio; 95% CI, 95% confidence intervals; SCC, squamous-cell carcinoma; AC, adenocarcinoma; Upp, upper esophagus; Mid, middle esophagus; Low, lower esophagus; BoM, bone metastases; BrM, brain metastases; LiM, liver metastases; LuM, lung metastases.

## Discussion

Due to the gradual popularity of screening for upper digestive tract cancers, the incidence of early local esophageal cancer keeps increasing in Asian countries (10, 11). A similar trend is also observed in the United States (3, 12). T1-2N0M0 and T1-2N0M1 EC are two extremes with completely different treatment strategies and survival outcomes. Therefore, the correct judgement of DM status in newly diagnosed T1-2N0 patients is of prime importance.

The majority of existing literatures focused on the risk factors identification of regional lymph nodes metastases (13–15). Some studies reported prognostic factors for metastatic EC, but didn’t explore factors associated with the occurrence of DM (16–18). Researches seeking risk factors for DM in EC are less common, let al.one literatures concerning risk factors of DM in T1-2N0 EC patients (19).

In this study, based on a large cohort of 10979 T1-2N0 EC patients from SEER database, five independent risk factors for DM were identified. Metastatic patterns of EC were elucidated, and independent risk factors for BoM, BrM, LiM, LuM were determined. More importantly, a nomogram to predict the likelihood of DM in patients with T1-2N0 EC was established with a good predictive accuracy. The variables included in the nomogram were extremely easy to obtain clinically. Via matching the parameters according to the nomogram, the risk of DM was easily calculated, thus helping doctors to determine whether to adopt further imaging examinations. Our study was significant with meaningful results and pragmatic function, promising in facilitating clinical decision-making in the future.

According to previous studies, the incidence of DM in superficial EC after endoscopic resection or esophagectomy ranges from 7% to 13% during follow-up (20, 21). Deng et al. reported a DM rate of 29.2% in newly diagnosed EC, but this study encompassed T3, T4 and N+patients (5). In our cohort, the DM rate in newly diagnosed EC was 12.1% (*n* = 561), representing a result from the largest known T1-2N0 EC cohort. The most common site of DM were liver (47.3%) and lung (27.0%), which was in line with that of previous reports (22, 23).

Among the six parameters incorporated in the nomogram, tumor size had the highest discriminating power. Moreover, in subgroup analysis, tumor size was significant associated with BoM, LiM, and LuM. These results were in line with that of previous studies, larger tumor size had been proven to be related to higher invasiveness and worse clinical outcome (24–26). In addition, tumor size had been identified as a predictor for DM in patients with EC in several previous researches (25–27).

Similarly, younger age at diagnosis was a well-acknowledged risk factor for several solid tumors (28). Previous studies repeatedly demonstrated that younger patients tend to show a more aggressive disease course (29). von Sochaczewski CO et al. and Lin YJ et al. found that EC patients of younger age were more prone to developing DM (27, 30). The phenomenon was also observed in our research, with age ≤60 possessing the highest DM risk. In subgroup analysis, age remained significant in predicting BoM and LiM. Concerning gender, Peng J et al. found that in rectal cancers, males were more likely to develop DM (31). However, the majority of existing literatures did not identify association between sex disparity and the risk of DM in EC (25–27). A few scholars found male gender associated with worse prognosis in EC patients (32). What’s more, von Sochaczewski CO et al. reported that male gender was in correlation with a higher risk of overall metastatic rate among patients with early EC (30). Sex-related differences in incidence of DM appeared to be of difficult explanation. The latent causes might be associated with gonadal hormone, tobacco use and socioeconomical factors. In the current study, male gender was related to higher risk of DM, and remained significant in subgroup analyses for LiM. Besides, a large number of studies repeatedly proved that poor differentiation indicated more aggressive biological behavior, leading to worse prognosis, as well as higher metastatic rate (30, 33). In this study, male gender and worse differentiation were significantly connected with higher DM probability. In subgroup analysis, probably owing to the large proportion of LiM, tumor grade merely remained significant in predicting LiM.

As for primary tumor site, we identified that tumor of lower esophagus possessed higher risk of DM (OR = 1.78, 95% CI 1.52–2.10, *P* < 0.01). In subgroup analysis, lower esophagus remained significant in predicting BoM and LiM. However, upper and middle esophagus were reported to be independently related to LuM. Based on existing literatures, it was widely published that tumor of lower esophagus was more inclined to present with DM, especially to liver (23, 34, 35). However, upper esophagus was reported to tend to develop LuM (35). As a result, the predictive significance of lower esophagus in overall cohort might be due to the large proportion of LiM, and subgroup analysis revealed the risk location of upper and middle esophagus for LuM. The anatomical hypothesis might explain this site associated metastatic disparity (35). The blood of upper and middle esophagus was drained through inferior thyroid vein and azygos vein, entering pulmonary circulation *via* superior vena cava. As for lower esophagus, blood was drained through left gastric vein, entering liver *via* portal vein.

According to a systemic literature review, DM was multiple in 46.5% cases (34). Several studies revealed that specific DM could increase the risk of DM in other anatomical sites (25, 33), a phenomenon was also observed in our research. The reason could be that lymphatic or hematogenous dissemination caused by metastatic sites might increase the occurrence of DM.

Finally, an interesting result was that T1 patients was significantly related to higher risk of DM compared to T2 patients. This seemed to go against common sense. As we know, higher T stage was independently associated higher incidence of regional lymph node metastases (13). As to the correlation between T stage and DM, however, there was no unanimity among scholars. Some of the existing literatures found there was no significant correlation (36). Several scholars found that greater tumor depth predict higher DM (30). On the contrary, Cheng S et al. found that T1 was the strongest risk factor LiM, followed by T4, T2 and T3 (37). Guo et al. found that T4 was the strongest risk factor LuM, followed by T1, T3 and T2 (9). Furthermore, Deng J et al. analyzed a cohort of 19,078 EC patients from SEER database and found that T1 stage predicted worse survival compared with T2 and T3 stage (5). In our view, the following reason might account for this phenomenon. Our study excluded cases with regional lymph node metastases. T2 stage was much more likely to have regional lymph node metastases than T1. As a result, the incorporated T2 cohort was more selective than T1, which meant that most of the biologically more aggressive T2 cases were excluded, leaving a T2 cohort of relatively less aggressive. Therefore, T1N0 patients were more likely to develop DM.

Despite the interesting discovery and practical nomogram of this study, it was essential to describe the limitations. Firstly, retrospective studies were inherently biased. Secondly, we didn’t know how the patients were clinically staged. We could not tell if the staging of primary tumor, regional lymph node, and tumor size were determined by esophageal endoscopic ultrasound or by CT. Thirdly, we didn’t know how distant metastases were confirmed or excluded. Were they all determined by PET-CT? What percentage of patients had a biopsy-proven DM? Moreover, distant lymph nodes and adrenal gland were also common metastatic stie of EC (7). Due to the information shortage, distant lymph nodes and adrenal gland were not described or put into statistical analyses.

## Conclusion

Our study presented as the largest series of T1-2N0 EC patients. The metastatic pattern of T1-2N0 EC was described for the first time. We identified independent predictive factors for DM, as well as for BoM, BrM, LiM and LuM. Above all, a practical and convenient nomogram with a great accuracy to predict DM probability for T1-2N0 EC patients was established. We hope our findings could facilitate clinical decision making in the future.

## Data Availability

The original contributions presented in the study are included in the article/Supplementary Material, further inquiries can be directed to the corresponding author/s.
